# Characteristic Gene Alterations During Fatty Acid Metabolism in the Goose Liver

**DOI:** 10.3390/genes16101137

**Published:** 2025-09-25

**Authors:** Anna Koseniuk

**Affiliations:** Department of Animal Molecular Biology, The National Research Institute of Animal Production, Krakowska 1 Street, 32-083 Krakow, Poland; anna.koseniuk@iz.edu.pl

**Keywords:** fatty acids, genetic background, goose, waterfowl

## Abstract

The development of hepatic steatosis in geese is a complex, multistage process involving genes related to lipid synthesis, transport, storage, and metabolism. Key genes activated during this process include ME1 (malic enzyme 1), SCD1 (stearoyl-CoA desaturase), ACSL1 (acyl-CoA synthetase long-chain family member 1), and ELOVL6 (elongation of very-long-chain fatty acids protein 6). The expression of these genes varies depending on the tissue, breed, and metabolic context. Geese possess a unique ability to develop hepatic steatosis (fatty liver) without accompanying inflammation or liver damage. This condition typically arises from overfeeding, either through carbohydrates or fats, leading to significant triglyceride accumulation in hepatocytes. Importantly, this state remains reversible and is considered non-pathological. The physiological and molecular changes observed in overfed geese, particularly regarding liver lipid accumulation and serum enzyme activity, closely resemble those found in human non-alcoholic fatty liver disease (NAFLD). This similarity makes geese an excellent biomedical model for studying NAFLD. Overfeeding initiates a cascade of enzymatic reactions that regulate lipid metabolism at the genetic level. These reactions decrease circulating free fatty acids and glucose while promoting triglyceride storage in the liver. The aim of this study is to synthesize current knowledge on the genetic regulation of fatty acid metabolism in geese, highlighting how these genes coordinate the processes of activation, desaturation, synthesis, and elongation during induced steatosis. Moreover, the summarized effects of different diet supplements will enhance goose feeding strategies for foie gras production.

## 1. Introduction

Lipogenesis is a key process in the energy storage system in mammals and other animals, including birds [1,2]. Geese possess a remarkable physiological ability to develop a fatty liver (hepatic steatosis) without adverse health effects. This condition is typically induced by excessive high-energy food (carbohydrates or fat) intake and is characterized by extensive lipid accumulation in the liver. Uniquely, this steatosis is reversible and non-pathological, distinguishing it from similar conditions in humans and other animals. The intense hepatic steatosis is similar to the development of non-alcoholic fatty liver disease (NAFLD) in humans and other mammals. Similarities in serum enzyme activity changes and liver lipid deposition mechanisms have been observed in overfed geese and humans or mammals with NAFLD [3]. As a consequence, goose fatty liver can be used as an excellent model for non-alcoholic fatty liver disease (NAFLD) in biomedical research [3,4].

This unique metabolic adaptation in geese is thought to be linked to their natural biology. It is widely believed that this reversible hepatic steatosis is an evolutionary adaptation linked to the migratory behaviour of geese. During long-distance flights, these birds must withstand prolonged periods of fasting, requiring efficient lipid storage and mobilization mechanisms [3].

To investigate the underlying mechanisms of this physiological trait, numerous studies have investigated changes in gene expression in overfed geese. Goose fatty liver forms primarily due to an imbalance between fatty acid synthesis, β-oxidation, lipid synthesis, and lipid transportation in the liver [2]. Overfeeding triggers hepatic steatosis accompanied by dynamic regulation of lipid metabolism-related genes, including both upregulated and downregulated factors.

Lipid metabolism plays a crucial biological role in living cells [5]. The studies conducted so far have demonstrated that lipid metabolism is crucial for numerous biological processes, such as reproduction (oocyte maturation and follicular development) [6].

This manuscript summarizes and organizes the systematic knowledge about the genetic background of the metabolism of fatty acids in geese. These genes play crucial roles in fatty acid activation, desaturation, synthesis, and elongation, and their regulation reflects the liver’s metabolic adaptation during induced steatosis. Moreover, the summarized effects of different diet supplements will enhance goose feeding strategies for foie gras production.

## 2. Materials and Methodology

A search for literature on gene alterations during fatty acid metabolism in goose liver was conducted on the Web of Science, Google Scholar, and PubMed databases. The following keywords were used: goose, diet supplementation, liver injury, liver fibrosis, NAFLD, and gene alteration. Emphasis was placed on articles published since 2018, but earlier articles were also included. Studies included original research as well as reviews in English that contained information about liver disease.

Table 1 provides a summary of fatty acid metabolism, accompanied by relevant references and information on the breeds examined.

## 3. Liver Fatty Acid Composition and Genetic Response to Different Diet Supplementation Types

There is a growing epidemic of nutrition-related diseases in highly developed societies. Excessive consumption of saturated fats is a key factor contributing to elevated blood cholesterol levels, which, over time, increases the risk of cardiovascular events such as strokes and heart attacks. Furthermore, a high intake of saturated fatty acids (SFAs) is strongly associated with metabolic disorders, including type 2 diabetes and non-alcoholic fatty liver disease (NAFLD) [4]. Current dietary guidelines recommend limiting the intake of SFAs, particularly those found in red meats such as pork and beef. Instead, it is advised to increase the consumption of food rich in monounsaturated (MUFAs) and polyunsaturated fatty acids (PUFAs). The essential fatty acids omega-3 and omega-6 are good examples of PUFAs, as they cannot be synthesized de novo and must be obtained through the diet. In this context, goose meat and livers, available on the market as foie gras, are the compelling alternatives. Studies on native Polish goose breeds have shown that goose meat is notably rich in PUFAs. Specifically, it contains high levels of n-6 fatty acids such as linoleic acid (C18:2 n-6), arachidonic acid (C20:4 n-6), and docosatetraenoic acid (C22:4 n-6), with concentrations in ranges of 13.20–16.20%, 3.61–7.79%, and 0.55–0.88% of total fatty acids, respectively. In terms of n-3 fatty acids, the meat contains linolenic acid (C18:3 n-3), eicosapentaenoic acid (EPA, C20:5 n-3), and docosahexaenoic acid (DHA, C22:6 n-3) at levels of 0.87–1.63%, 0.58–1.51%, and 0.34–0.44%, respectively [21]. The fatty livers obtained from Landes geese are rich in MUFAs and PUFAS [3,7] (Fournier et al. 1997, Zhu et al. 2011). For optimal health, the balance between n-6 and n-3 fatty acids is critical, with an ideal dietary ratio close to 1:1 [22]. Research on Polish goose breeds indicates that both the n-6:n-3 ratio and the overall fatty acid profile are influenced by rearing conditions, including diet composition and genetic background [8,10].

The vast majority of research has focused on the alteration of fatty acid composition induced by overfeeding. Overfeeding is the procedure of providing a goose or duck a hefty dose of high-energy food (carbohydrates or fat). Under the Council Directive 98/58/EC, which sets the minimum standards for the welfare of farmed animals, Article 14 stipulates that animals must be fed in a manner that avoids causing unnecessary suffering. Due to this legal framework, forced overfeeding of geese and ducks is not commonly practiced across the majority of European countries. However, it remains legally permitted in France, Hungary, Bulgaria, Spain, and Wallonia, a region of Belgium.

France stands out as a global leader in the production of foie gras, which is known for its exquisite delicacy and unparalleled flavour [11]. The French Grey Landes goose has emerged as the predominant breed, prized for its remarkable ability to accumulate triglycerides in the liver and effectively increase liver weight [3]. In contrast, Asia has developed a burgeoning market for breeding European-origin Landes geese, signalling a growing international trend in this industry [12].

The overfeeding results in an excessive amount of energy being provided, inducing fatty acid alteration. These processes support the reversible, non-pathologic accumulation of triglycerides in goose liver. In short, it is induced by enhancing membrane flexibility and lipid storage. All of this is happening simultaneously to limit inflammatory damage [2].

The genetic response to overfeeding is induced differentially depending on the energy source used. Overfeeding induces the accumulation of triglycerides in hepatocytes; however, different pathways related to protein transport, hepatocyte proliferation, and fatty acid composition, including essential fatty acids, are also induced. Feeding geese with carbohydrates and dietary lipids (oils/fats) triggers distinct metabolic processes in their livers, even though both lead to hepatic steatosis [2,13]. The genetic mechanisms remain nutritionally modulated, although the fatty acid content differs slightly. A precise and thorough analysis of the effects of different dietary components in geese allows for a critical evaluation of fatty acid concentration and composition. This, in turn, enables improvements in foie gras production methods, encouraging producers to consider not only performance parameters but also to remain aware of product quality, while ensuring that geese are maintained in optimal health throughout the fattening process.

A detailed study explored the differences in lipid deposition mechanisms in goose liver at the individual, transcriptome, and cellular levels by comparing the metabolic pathways induced by overfeeding with maize [7,14], glucose, sucrose, and fructose [2]. High-energy carbohydrates are commonly used in livestock production. A higher intake of carbohydrates induces an increase in liver lipids and is positively correlated with de novo fatty acid synthesis, as it is believed that glucose, fructose, or amino acids are considered the primary de novo fatty acid synthesis substrates [2,23].

In geese overfed carbohydrates, fructose, glucose, and sucrose, intracellular triglycerides (TGs) increased, and noticeable lipid deposition was observed in the primary hepatocytes of the studied geese [2]. The genetic alteration induced by carbohydrate supplementation is summarized in Table 2. The dietary addition of glucose has a potentially suppressive effect on hepatic inflammation. In [15], the primary hepatocytes were treated with glucose, which increased the ubiquitination level of PKA, which are proteins associated with various chronic diseases, such as NAFLD [15]. Moreover, in the liver tissues of the group treated with fructose, glucose, and sucrose, the expression of the genes related to fatty acid synthesis (fatty acid synthetase (FAS) and acetyl-CoA carboxylase (ACCa)) increased. However, this mechanism was not proven in [13]. Interestingly, the expression of FABP1, a gene related to lipid transport, is decreased in the sucrose groups. In this group, a significantly higher expression of the PPAR signalling pathway was observed, which stimulates lipid metabolism and fat cell differentiation. These molecular mechanisms were reflected in the highest-weight livers in the sucrose overfeeding group [2]. In a later study, overfeeding with supplementation of 10% fructose did not induce the expression of genes of the key enzymes involved in hepatocyte fatty acid synthesis and steroid biosynthesis (ELOVL6, SCD, FASN, and SREBP1) [13]. Anti-inflammatory mechanisms typically activated during the process of lipid accumulation in the liver, protecting the liver from severe hepatic steatosis, were also not induced in [13]. According to the authors of [13], the procedure of overfeeding with fructose supplementation was maintained for too long. Therefore, after fat accumulation, the expression of genes involved in hepatocytes’ lipid synthesis was downregulated, and the lipid accumulation slowed down [13]. The natural ability of waterfowl to accumulate lipids in the liver is, as mentioned, reversible, and effective anti-inflammatory processes are likely time-limited [13]. Based on previous studies, there is likely a specific time range in which the anti-inflammatory mechanisms are induced. This is closely associated with the increase in key genes involved in hepatocyte fatty acid synthesis and steroid biosynthesis.

The type of sugar added induces changes in fatty acid profiles in the liver. The sugar addition increased the content and proportion of UFAs (unsaturated fatty acids). The concentration and the ratio of essential n-3 and n-6 PUFAs were also higher in the sugar overfed groups [2]. However, the geese overfed with maize flour supplemented with 10% fructose expressed remarkable liver steatosis [13].

In the production of foie gras, the most effective supplementation method involves sucrose. The addition of sucrose leads to the development of heavier livers, positively impacting economic returns. Although glucose supplementation induces anti-inflammatory processes most effectively during short-term overfeeding (for a duration of several weeks), the non-inflammatory effects of sucrose supplementation will still be adequate.

The studies elucidating the effect of fat (either animal or plant) supplementation on fat synthesis and triglyceride accumulation in goose liver are in the minority. In [11], geese in the overfeeding group received an additional dose of plant oil, specifically soybean oil, goose fat, and a mix of both these fat types. The addition of rapeseed oil and goose fat was tested by [16]. Both groups studied Landes geese. In [11], birds were sacrificed at 60 days of life, while in [16], they were sacrificed at 70 days of life. Ref. [16] proved that the concentration of omega-6 PUFAs increased, while the levels of saturated fatty acids (SFAs) were lower in the group fed with rapeseed oil compared to the goose fat group. Concentrations of long PUFAs, specifically C16:1, C18:1, C18:2, C20:2, and C22:1, were higher in the rapeseed oil group [16]. There was no statistically significant difference in the concentration of omega-3 fatty acids between the groups. Notably, the ratio of n-3 to n-6 PUFAs was substantially lower in the fatty liver of both overfed geese relative to that of the control group [16]. In the study conducted by [11], the type of fat used did not affect the levels of saturated fatty acids (SFAs) or PUFAs, including omega-3 (n-3) and omega-6 (n-6) PUFAs. However, the group supplemented with soybean oil had the lowest concentration of unsaturated and monounsaturated fatty acids (MUFAs) in the liver when compared to the groups receiving goose fat and mixed-fat supplements [11]. The results suggest that when considering fat supplementation in a goose’s diet, it is more beneficial to choose rapeseed oil over soybean oil and goose fat. This choice leads to foie gras that is richer in both omega-6 polyunsaturated fatty acids (PUFAs) and long-chain PUFAs. On the other hand, overfeeding geese with a mixture of goose fat and rapeseed oil is more economically advantageous, as it results in a higher liver weight. However, it is worth noting that there is a 10-day difference in the collection time of the studied material, which might impact the final results obtained.

Several common genes involved in fat metabolism differentiation, as well as oxidative stress, have been identified as DEGs between the goose fat and rapeseed oil-fed and soybean oil-fed groups (Table 3). Notably, both studies revealed distinct profiles of significantly differentially expressed genes between the treatment groups, with the exception of the SCD gene, which consistently emerged as a commonly regulated target across both studies. Ref. [16] found that ELOVL1, ELOVL2, and ELOVL3 were downregulated. These genes belong to the large group of genes involved in fatty acid elongation. ELOVL1 is primarily involved in the elongation of saturated and monounsaturated fatty acids (C20–C26). In contrast, ELOVL2 plays a significant role in elongating polyunsaturated fatty acids (PUFAs), particularly by converting C20 and C22 PUFAs into C24 precursors that are necessary for the biosynthesis of docosahexaenoic acid (DHA) and other long-chain PUFAs. Additionally, the ELOVL3 gene also contributes to the elongation of saturated and monounsaturated fatty acids. Notably, all of the ELOVL-type genes that were identified are downregulated in both studied groups, which may suggest that hepatocytes experience a high accumulation of polyunsaturated fatty acids (PUFAs). The SCD (stearoyl-CoA desaturase) gene was upregulated with a higher concentration in the group fed goose fat [16]. In the study by [11], this gene was downregulated, similar to ELOVL6, FASN, and SREBP1. The observed effect is likely a consequence of the extended overfeeding duration, during which hepatic steatosis had already been fully established, leading to a downregulation of pathways associated with fatty acid biosynthesis and elongation. It is compelling that the genetic machinery related to fatty acid elongation in a group fed with goose fat does not result in a higher concentration of long fatty acids in the livers affected by steatosis. This mechanism is likely less efficient compared to the transformation of plant oils.

## 4. Genetic Response to Hepatic Steatosis

The development of hepatic steatosis in geese induced with supplementation with carbohydrates is a complex, multistage process requiring the involvement of numerous genes associated with lipid synthesis, packaging, secretion, transport, deposition, and metabolism [24]. The biosynthesis of fatty acids in goose liver is catalyzed sequentially by enzymes that are reflected in the activation of key genes during the reversible process of hepatic steatosis in geese, including ME1 (malic enzyme 1), SCD1, ACSL1 (acyl-CoA synthetase long-chain family member 1), and ELOVL6 (elongation of very-long-chain fatty acids protein 6). However, these genes show differential expression patterns depending on tissue, breed, and metabolic context [1].

Among these, SCD1 is consistently upregulated during overfeeding-induced steatosis and plays a central role in lipogenesis. SCD1 catalyzes the conversion of saturated fatty acids (SFAs) such as palmitoyl-CoA (C16:0) and stearoyl-CoA (C18:0) into their monounsaturated counterparts by introducing a cis-double bond at the delta-9 position [24,25,26]. The resulting monounsaturated fatty acids (MUFAs) serve as major substrates for the biosynthesis of complex lipids such as triglycerides, phospholipids, wax esters, and cholesterol esters, and play a critical role in maintaining cell membrane fluidity [24].

**Figure 1:**
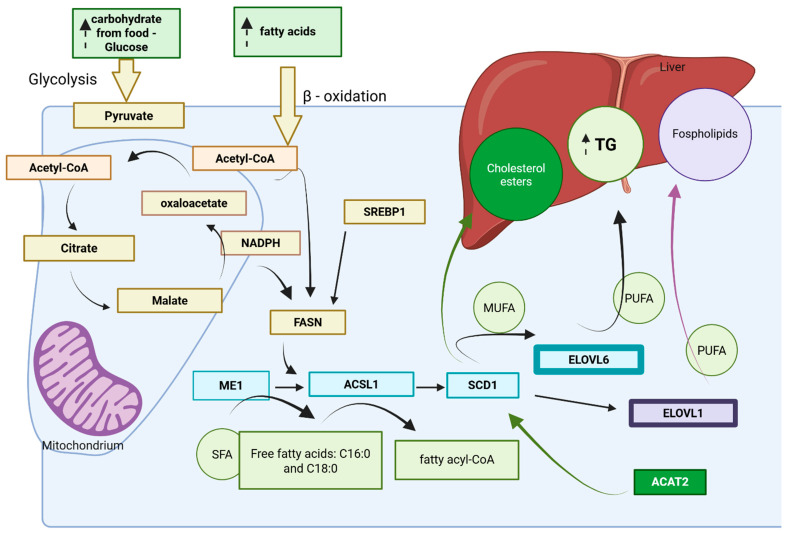
The schematic genetic regulation pathways induced with de novo FA substrates increasing the formulation of triglycerides, phospholipids, and cholesterol esters in the goose, induced with supplementation with carbohydrates; glucose and free fatty acids enter the hepatic cytosol, beginning glycolysis and providing Acetylo-CoA, an essential substrate for the citric acid cycle, which takes place in mitochondria; FASN (fatty acid synthase) activity is induced with SREBP1 (sterol regulatory element-binding protein 1); and FASN (belongs to the FAS enzymatic complex) catalyzes the de novo synthesis of palmitic acid (C16:0) from acetyl-CoA, using NADPH as a reducing equivalent. It acts as the entry point into the lipid synthesis network, providing saturated fatty acids (SFAs) that serve as substrates for downstream elongation and desaturation. FASN activity is tightly linked to ME1; ACSL1 converts free fatty acids into fatty acyl-CoA esters, activating them for further elongation and desaturation or incorporation into TG (lipid storage purposes) and phospholipids (membrane synthesis). Blue boxes—genes upregulated during overfeeding; ME1—malic enzyme 1; ACSL1—acyl-CoA synthetase long-chain family member 1; SCD1—stearoyl-CoA desaturase 1. The activity of the genes marked with a wide frame is modulated with ACSL1 and SCD1 activity: ELOVL6 and ELOVL1—elongation of very-long-chain fatty acids protein 6 and 1, respectively; ACAT2 (Acyl-CoA:Cholesterol Acyltransferase 2)—with a dark green colour, marks the cholesterol esters’ synthesis pathway; ELOVL1—light purple colour, marks the phospholipids’ synthesis pathway; yellow boxes—substrates for glycolysis, biosynthesis of unsaturated fatty acids, and triglyceride synthesis (created in https://BioRender.com).

Figure 1 presents a schematic overview of the genetic regulation of lipid biosynthesis in the goose liver under carbohydrate overfeeding.

SCD1 expression is tightly regulated in response to nutritional and hormonal cues and is localized to the endoplasmic reticulum, where it undergoes rapid turnover [27]. Comparative genomics reveals that the goose genome harbours more than three times as many SCD gene copies as *Gallus gallus*, *Anser cygnoides*, or *Homo sapiens*, which may underlie its exceptional lipogenic capacity [25]. The expression of SCD1 is known to be promoted by insulin and inhibited by leptin, influencing triglyceride accumulation in hepatocytes [28]. Additionally, SCD1 is regulated by SREBP-1c and can also respond to changes in the levels of saturated fatty acids [29]. While SCD1 is broadly expressed, it shows the highest levels in liver and adipose tissues and much lower expression in the spleen, where downregulation may play a protective, anti-inflammatory role post-overfeeding [24,30].

SCD belongs to the fatty acid desaturase (FADS) family, which catalyzes critical steps in the conversion of SFAs to MUFAs (Moltó-Puigmartí et al., 2010) [31]. Osman et al. (2016) [32] identified FADS1 and FADS2 as protective elements during goose hepatic steatosis, which were upregulated to meet the immediate demand for long-chain polyunsaturated fatty acids (LC-PUFAs), thereby preventing liver injury. Similarly, Loix et al. (2024) [33] showed that the steady expression of SCD1 in the hypothalamus helps prevent inflammation caused by elevated circulating fatty acid levels.

The role of ELOVL6, a long-chain fatty acid elongase, is also crucial in this metabolic network. It works in conjunction with FAS and SCD1 in the de novo synthesis of saturated and monounsaturated fatty acids. SREBP1 regulates ELOVL6 expression, and its sustained activation induces overexpression of nuclear SREBPs in hepatocytes [34]. SREBPs exist in three isoforms—SREBP1a, SREBP1c, and SREBP2—each governing distinct aspects of lipid metabolism. SREBP2 is mainly involved in cholesterol biosynthesis, whereas SREBP1c regulates adipogenesis by modulating its transcriptional levels.

In geese, dietary manipulation—especially overfeeding with carbohydrate-rich diets—is the primary strategy used to induce hepatic steatosis. Glucose and other monosaccharides act as substrates for de novo lipogenesis and simultaneously stimulate the expression of ME1, which generates NADPH, a necessary cofactor for fatty acid biosynthesis [7,35]. Unlike mammals, geese heavily rely on NADP+-dependent malic enzyme activity for this process [36]. The authors of [9] observed significant breed-specific variation in ME1 expression, with Landes geese showing higher expression compared to the Kielecka and White Koluda^®^ breeds, suggesting a genetic predisposition toward enhanced lipogenesis in the Landes breed.

Overfeeding also increases hepatic expression of ACSL1, ELOVL5, and SCD1 [7]. ACSL1 activates long-chain fatty acids by converting them into fatty acyl-CoA, which are then elongated by ELOVL6 and desaturated by SCD1. The ACSL1 and ELOVL6 genes display intriguing expression patterns. ACSL1 is involved in the uptake and storage of fatty acids, with its expression significantly increasing during periods of overfeeding. ELOVL6 is also crucial for the elongation of fatty acids while also preventing excessive elongation that could lead to lipid toxicity in cells [17,37]. The opposing expression patterns of these two genes help to balance the types of lipids that are synthesized and stored, thereby preventing the harmful buildup of long-chain saturated fatty acids [17]. This results in the production of very-long-chain unsaturated fatty acids, which are essential for membrane biosynthesis, energy storage, and signalling [7,25]. The authors of [9] also showed higher ACSL1 expression in Landes geese compared to local Polish breeds, further reinforcing the notion of genetic specialization for fatty acid metabolism.

In addition to these genes, FASN plays a central role in de novo lipogenesis, being highly expressed in goose liver and directly controlling fatty acid synthesis [12]. FAS is a multifunctional enzyme complex that catalyzes the terminal steps of de novo fatty acid synthesis, converting acetyl-CoA and malonyl-CoA into palmitate (C16:0) using NADPH as a reducing agent. In the goose liver, FAS is highly expressed under overfeeding conditions—especially with carbohydrate-rich diets—facilitating increased lipid deposition [12]. The activity of FAS is closely coordinated with upstream genes such as ME1 (malic enzyme 1), which supplies NADPH, and downstream enzymes like ELOVL6 and SCD1, which elongate and desaturate fatty acid chains. The upregulation of FAS in overfed geese is essential for hepatic triglyceride synthesis, providing the bulk of saturated fatty acid substrates required for further elongation and lipid storage (Figure 1).

Other relevant enzymes include ACAT2, involved in cholesterol ester synthesis, and ELOVL1, which participates in PUFA elongation [32]. ELOVL1, part of the same elongase family as ELOVL6, plays a complementary role (Figure 1). ELOVL1 plays a pivotal role in the elongation of saturated and monounsaturated fatty acids with 20 to 26 carbon atoms, generating very-long-chain fatty acids (VLCFAs) that are crucial for the integrity of membrane phospholipids, sphingolipids, and lipid signalling molecules. In the context of hepatic steatosis, ELOVL1 operates downstream of fatty acid synthase (FAS), extending the fatty acid chains initially produced by FAS. This elongation is not just important but essential for maintaining membrane fluidity and ensuring that cellular organelles in lipid-rich hepatocytes possess the structural and functional properties necessary for optimal performance.

Furthermore, research by [12] highlights the critical function of ACAT2 in the esterification of free cholesterol with long-chain fatty acyl-CoA, resulting in the formation of cholesterol esters (CEs) that are vital for storage and secretion (Figure 1). In overfed goose liver, the upregulation of ACAT2 is significant, particularly when diets are enriched with fat-rich components like goose fat or mixed lipids. This underscores the biochemical adaptations that occur in response to dietary changes, revealing the intricate balance of lipid metabolism in the liver [12,32]. These genes are differentially expressed in geese depending on the type of dietary fat administered, with mixed-fat diets (e.g., plant oil + goose fat) enhancing expression and promoting greater retention of unsaturated fatty acids [11].

## 5. Epigenetic Regulation of Gene Expression Involved in Hepatic Lipid Biosynthesis in Geese

Epigenetic modifications, i.e., DNA methylation, histone modifications, and regulation by non-coding RNAs (miRNAs, lncRNAs, and circRNAs), play pivotal roles in regulating gene expression without altering the DNA sequence. These modifications allow the goose liver to adapt flexibly to environmental and nutritional changes during hepatic steatosis. In geese, understanding epigenetics is particularly relevant because their liver serves as the central site for lipogenesis, unlike mammals, where adipose tissue dominates fat storage. Studying these modifications in goose liver is crucial to uncover how reversible hepatic steatosis is orchestrated at the molecular level and to inform both poultry production and human NAFLD models [17,18].

Gene activity in goose hepatic lipid metabolism is tightly modulated through several layers of epigenetic regulation. Ref. [17] developed a microRNA (miRNA) profile in the livers of geese. They identified differentially expressed miRNAs, predicted their target genes, and categorized them based on the KEGG pathway analysis. Many of these target genes are integral to metabolic pathways that contribute to the development of hepatic steatosis in goose fatty liver. ACSL1 and ELOVL6 are predicted target genes of aan-miR-203a and aan-miR-125b-5p, respectively. ACSL1 and ELOVL6 exhibited opposite expression patterns with aan-miR-203a and aan-miR-125b-5p, respectively. These findings suggest that both types of miRNA regulate hepatic lipid metabolism by targeting ACSL1 and ELOVL6 [17].

Ref. [19] conducted a comprehensive analysis of miR-29c and its predicted target genes, including Col3a1, Sgk1, and Insig1, suggesting that miR-29c may play a regulatory role in energy homeostasis and cell growth. The miR-29c suppression in energy metabolism-related tissues of overfed geese indicates that miR-29c could be involved in the regulation of energy balance during the development of fatty liver in geese. Ref. [20] identified over 11,000 circRNAs and nearly 1000 miRNAs in goose liver, with many enriched in lipid metabolism pathways, such as fatty acid biosynthesis and PPAR signalling. Regulatory axes such as circRNA1112–miR-27b–target mRNA provide examples of ceRNA mechanisms modulating gene expression epigenetically during liver development and overfeeding.

Ref. [5] identified over 300 differentially expressed lncRNAs in the goose liver after overfeeding, with lncRNA XLOC_292762 notably downregulated, suggesting roles in controlling lipid accumulation and energy balance. Additionally, ref. [38] demonstrated that methylation of the VNN1 gene, a regulator of coenzyme A synthesis, was modulated by betaine and 5-azacytidine treatments, thereby influencing lipid synthesis in goose liver.

Histone modifications were not directly measured in most of the studies, but transcriptional changes in key metabolic and protective genes (e.g., HSP70, FADS1, and FABP) suggest upstream epigenetic regulation in response to oxidative stress during hepatic steatosis in a study conducted on Lion-Head Geese [18]. Moreover, it is believed that there is a coordinated regulation involving mRNA–lncRNA–circRNA networks during high-energy intake, with key metabolic enzymes like ME1 and PKS under an epigenetic regulation mechanism [18].

Hepatic steatosis in geese is modulated by a complex interplay of genetic and metabolic responses, with epigenetic regulators acting as key intermediaries. The miRNA–mRNA interactions (e.g., miR-125b–ELOVL6), circRNA networks, and DNA methylation effectively modulate the hepatic lipid profile. Genes such as SCD1, ACSL1, ELOVL6, ME1, and FASN are not only transcriptionally upregulated during overfeeding but also regulated in post-transcriptional processes. Interestingly, while hepatic steatosis in mammals often leads to pathological consequences, in geese, it remains largely non-inflammatory and reversible—a fact partly attributed to their unique epigenetic responses [5,19].

Non-coding RNAs, such as lncRNAs, miRNAs, or circRNAs, are promising therapeutic targets due to their roles in various cellular processes and their disease-specific expression patterns. The NAFLD-like phenotype in goose liver that develops without inflammation is promising for the development of therapeutic pathways in humans.

## 6. Conclusions

Carbohydrate supplementation tends to lower SFAs and increase MUFAs and PUFAs, suggesting a more favourable fatty acid profile in the liver, which is more preferable for the human diet. Fat supplementation shows the highest MUFA levels, particularly oleic acid, and maintains PUFA levels similar to those of controls. The omega-6 and omega-3 levels are relatively stable across all groups, but slightly lower with fat supplementation. During overfeeding, geese exhibit a coordinated upregulation of lipid metabolic genes—FASN → ACSL1 → ELOVL6 → SCD1—to drive efficient de novo lipogenesis, culminating in hepatic fat accumulation. This synchronized enzymatic cascade underpins the metabolic basis for reversible fatty liver formation in waterfowl.

In the future, foie gras producers will be able to rely on a range of evidence-based recommendations derived from current research findings. First, the duration of feeding and the type of dietary supplements used are crucial factors. These elements indicate to producers that a fatty liver contains a variety of valuable and nutritionally desirable polyunsaturated fatty acids (PUFAs) and monounsaturated fatty acids (MUFAs). Additionally, it is important to determine the optimal feeding period while ensuring the health and well-being of the birds, particularly by supporting anti-inflammatory processes.

Research on epigenetics and post-transcriptional modifications holds significant potential for advancing therapeutic strategies for the treatment of non-alcoholic fatty liver disease (NAFLD) in humans.

## Figures and Tables

**Table 1:** Summarized data on fatty acid metabolism with corresponding references and studied breeds.

Reference	Fatty Acid Profiles	Transcriptome/Gene Study	Epigenetic Modifications	Protein/Amino Acid Profiles	Repository/Repositories and Accession Number(s) *	Breed
[2]	y	y	n	y	https://figshare.com/s/70fbe6ff915e6471c431 (accessed on 10 September 2025); https://international.biocloud.net/zh/dashboard (accessed on 10 September 2025); ID:15215045770; Password: deng19940227	Tianfu
[3]	y	n	n	n	nd	Landes (France)
[7]	n	y	n	n	GenBank Accession no. GW713791-GW713889 and GW714091-GW714097; GenBank Accession no. HO224431 to HO224443	Landes (China)
[8]	y	n	n	n	nd	Kartuska, Lubelska
[9]	n	y	n	n	nd	Kielecka, Landes (Poland), and White Koluda®geese
[10]	y	n	n	n	nd	White Koluda®geese
[11]	y	y	n	y	https://doi.org/10.1016/j.aninu.2025.03.003.	Landes (China)
[12]	y	y	n	n	nd	Xupu
[13]	y	y	n	n	https://figshare.com/articles/dataset/Integrative_Analysis_between_Transcriptome_and_Lipidome_Reveal_Fructose_Pro-Steatosis_Mechanism_in_Goose_Fatty_Liver_Formation/21060628/1 (accessed on 10 September 2025).	Tianfu
[14]	y	y	n	y	10.6084/m9.figshare.21154540.	Tianfu
[15]	n	y	y	y	nd	Landes (China)
[16]	y	y	n	n	https://doi.org/10.1016/j.gene.2018.05.122.	Landes (China)
[17]	n	n	y	n	NCBI (GEO99200, https://www.ncbi.nlm.nih.gov/geo/query/acc.cgi?acc=GSE99200, accessed on 10 September 2025).	Landes (China)
[18]	n	y	y	n	Gene Expression Omnibus (GEO) Accession No. GSE243829	Lion-head goose
[19]	n	n	y	n	nd	Landes (China)
[20]	n	n	y	n	China National Center for Bioinformation Accession No. CRA014346 and CRA012842; https://www.mdpi.com/article/10.3390/ani14060839/s1 (accessed on 10 September 2025)	Sichuan white goose

* The repository/repositories and accession number(s) to raw data and results of sequencing, transcriptome, metabolism, and protein analysis; y—yes, the data were included in the manuscript; n—no, the data were not included in the manuscript; nd—no data.

**Table 2:** The up- and downregulated genes in the livers of geese overfed with carbohydrates (maize, glucose, fructose, and sucrose).

Gene Name	Supplementation Type, Reference, and Breed Studied			
	Maize	Reference/Goose Breed	G, F, S	Reference/Goose Breed
ELOVL6	up	Tianfu [14] Landes [7]	down (G, F, S)	Tianfu [2]
SCD1	up		nd	
ACSL1	up		nd	
ME1	up		nd	
FAS	down		up	
FABP	nd		up (G, F)	
PPAR	nd		up (F, S)	

G—glucose; F—fructose; S—sucrose; nd—no data.

**Table 3:** Genes involved in fatty acid metabolism, up- and downregulated in the livers of geese overfed with fat (goose fat or plant oil).

Gene Name	Supplementation Type, Reference, and Breed Studied	
	Goose Fat, Rapeseed Oil [16]	Soybean Oil [11]
ELOVL1	down	nd
ELOVL2	down	nd
ELOVL3	down	nd
SCD	up	down
ACACA	up	nd
SLC2A2	up	nd
SLC2A5	up	nd
SLC5A9	up	nd
ACSBG2	up	nd
FASN	nd	down
ELOVL6	nd	down
SREBP1	nd	down
LPL	nd	up

nd—no data.

## Data Availability

No new data were created or analyzed in this study. Data sharing is not applicable to this article.
